# Acute eGFR Changes With Finerenone Examined in a Prespecified Analysis of FINE-ONE

**DOI:** 10.1016/j.ekir.2026.107083

**Published:** 2026-09-16

**Authors:** David Z.I. Cherney, Tae Won Yi, Hiddo J.L. Heerspink, Linong Ji, Janet B. McGill, Peter Rossing, Katherine R. Tuttle, Jesper N. Bech, Paolo Fiorina, Albert Power, Karoline Schousboe, Nicholas M. Selby, Emanuela Setola, J. David Smeijer, Robert Lawatscheck, Meike Brinker, Julie Russell, Patrick Schloemer, Per-Henrik Groop, FINE-ONE Investigators

**Affiliations:** 1 Division of Nephrology, University Health Network, Toronto General Hospital, University of Toronto, Toronto, Ontario, Canada; 2 The George Institute for Global Health, University of New South Wales, Sydney, Australia; 3 Department of Clinical Pharmacy and Pharmacology, University of Groningen, University Medical Center Groningen, Groningen, Netherlands; 4 Department of Endocrinology and Metabolism, Peking University People’s Hospital, Peking University Diabetes Center, Beijing, China; 5 Division of Endocrinology, Metabolism & Lipid Research, Washington University in St. Louis, School of Medicine, St. Louis, Missouri, USA; 6 Steno Diabetes Center Copenhagen, Herlev, Denmark; 7 Department of Clinical Medicine, Faculty of Health and Medical Sciences, University of Copenhagen, Copenhagen, Denmark; 8 Providence Medical Research Center, Providence Inland Northwest Health, Spokane, Washington, USA; 9 Nephrology Division and Kidney Research Institute, University of Washington School of Medicine, Seattle, Washington, USA; 10 Department of Clinical Medicine, Aarhus University, Aarhus, Denmark; 11 University Clinic in Nephrology and Hypertension, Gødstrup Hospital, Herning, Denmark; 12 Division of Endocrinology, ASST Fatebenefratelli-Sacco, Milan and Università degli Studi di Milano, Milan, Italy; 13 Population Health Sciences, Bristol Medical School, University of Bristol, and Renal Unit, North Bristol NHS Trust, Bristol, UK; 14 Steno Diabetes Center Odense, Odense University Hospital, Odense, Denmark; 15 Centre for Kidney Research and Innovation, Academic Unit for Translational Medical Sciences, Royal Derby Hospital Campus, University of Nottingham, Derby, UK; 16 Cardio-metabolic and Clinical Trials Unit, IRCCS Ospedale San Raffaele, Milan, Italy; 17 Cardiology and Nephrology Clinical Development, Bayer AG, Berlin, Germany; 18 Cardiology and Nephrology Clinical Development, Bayer AG, Wuppertal, Germany; 19 Bayer PLC, Reading, UK; 20 Clinical Statistics and Analytics, Bayer AG, Berlin, Germany; 21 Department of Nephrology, University of Helsinki and Helsinki University Hospital, Helsinki, Finland

**Keywords:** chronic kidney disease, eGFR, finerenone, glomerular filtration rate, nonsteroidal mineralocorticoid receptor antagonist, type 1 diabetes

## Abstract

**Introduction:**

The nonsteroidal mineralocorticoid receptor antagonist finerenone reduces urine albumin-to-creatinine ratio (UACR) in people with type 1 diabetes (T1D) and chronic kidney disease (CKD). This prespecified analysis evaluated acute changes in estimated glomerular filtration rate (eGFR) with finerenone and their impact on safety.

**Methods:**

Adults with T1D and CKD (*n* = 242; eGFR ≥ 25–< 90 ml/min per 1.73 m^2^; UACR ≥ 200 to < 5000 mg/g) receiving stable renin-angiotensin system (RAS) inhibition were randomized 1:1 to finerenone or placebo. The primary outcome was acute change in eGFR (from baseline to month 1), termed “eGFR dip.” Reversibility of eGFR change was assessed from end of the treatment (month 6) to follow-up (month 7). Change in eGFR was assessed across eGFR dip subgroups (eGFR > 0 to ≤ 10% decline, > 10% decline, and ≥ 0% increase) and prespecified characteristics (baseline eGFR, UACR, history of cardiovascular disease [CVD], and diuretic use).

**Results:**

Mean acute eGFR decline at month 1 was greater with finerenone (−2.78 ml/min per 1.73 m^2^ [95% confidence interval [CI], −4.15 to −1.40]) versus placebo (−0.78 ml/min per 1.73 m^2^; [95% CI, −2.22 to 0.66]); treatment difference was –1.99 ml/min per 1.73 m^2^ (95% CI, –3.97 to –0.01 [*P* = 0.049]). Placebo-corrected eGFR increased during the washout period across the groups. Acute eGFR changes did not differ according to prespecified characteristics. The incidence of treatment-emergent adverse events (TEAEs) was comparable across eGFR dip subgroups in both the treatment groups. Greater declines in eGFR were associated with greater reductions in UACR at month 3.

**Conclusion:**

In people with T1D and CKD, finerenone induced an acute eGFR dip that was well tolerated without unexpected safety signals.

## Graphical abstract

**Figure:**
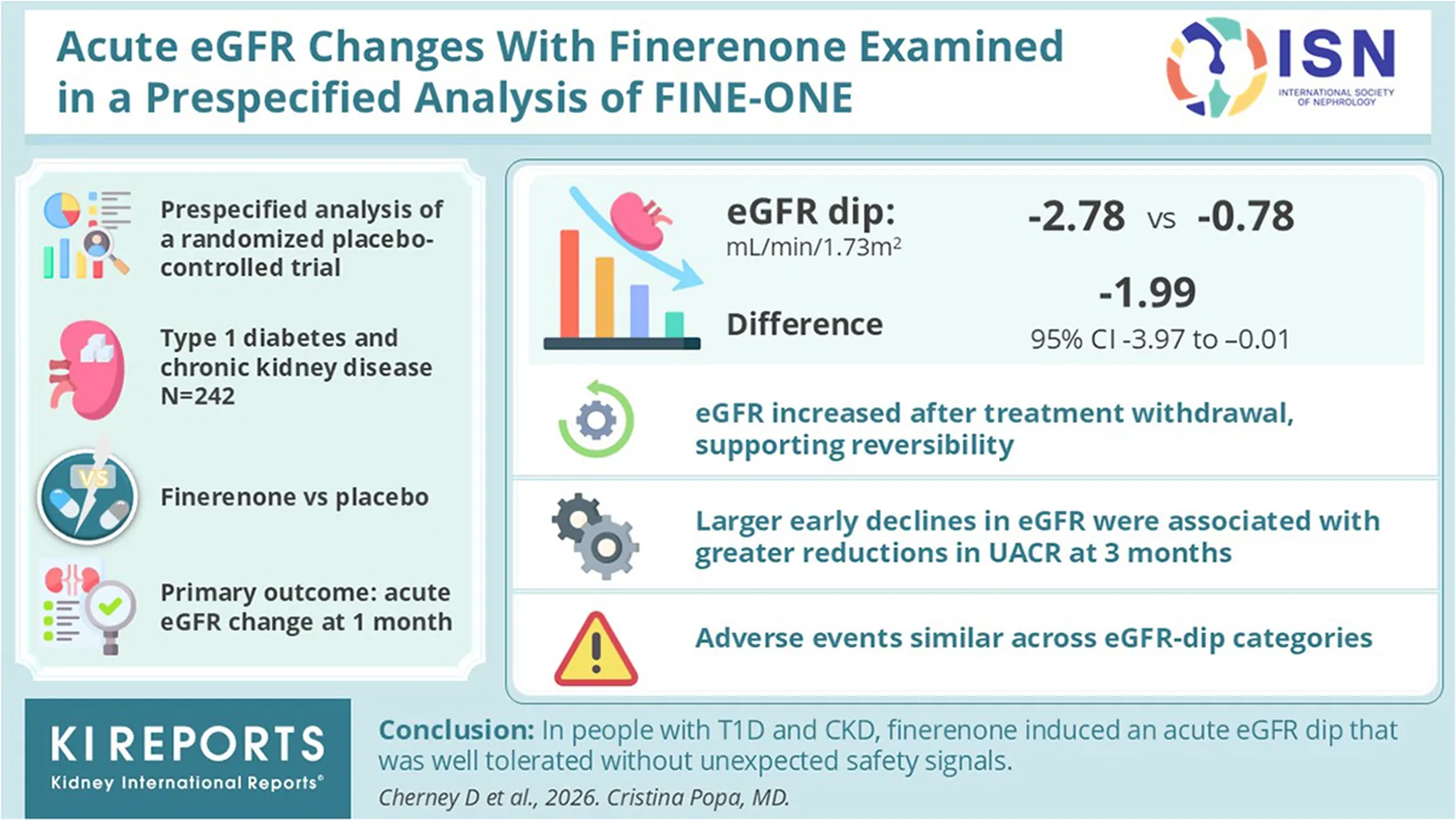

T1D is a major risk factor for the initiation and progression of CKD, including kidney failure. CKD also promotes CVD and increases the risk of mortality.^1^, ^2^, ^3^ Over the past 30 years, CKD treatment in people with T1D and type 2 diabetes (T2D) has focused on glycemic and blood pressure control, the latter especially with RAS inhibitors primarily to attenuate glomerular hypertension.^4^^,^^5^ In addition to RAS inhibitors, 3 pharmacologic therapies have been shown to preserve kidney function in people with T2D and CKD: sodium-glucose cotransporter-2 (SGLT2) inhibitors,^6^, ^7^, ^8^ the glucagon-like peptide-1 receptor agonist semaglutide,^9^ and the nonsteroidal mineralocorticoid receptor antagonist (nsMRA) finerenone,^10^ which act through complementary physiological mechanisms.^11^ Although these therapies have substantially advanced kidney protection in T2D, evidence in T1D remains limited, because clinical trials have largely excluded this population owing to safety concerns and the relative rarity of T1D.^12^, ^13^, ^14^ Consequently, individuals with T1D, particularly those with albuminuria,^15^, ^16^, ^17^ remain at risk for both CKD and CVD.^5^^,^^18^^,^^19^

The FINE-ONE trial was designed to evaluate the efficacy of finerenone on markers of kidney protection in people with T1D at high risk of CKD progression.^20^ In the FINE-ONE trial, finerenone lowered UACR by 25% over 6 months, showing an albuminuria-lowering effect in T1D consistent with that observed in T2D.^21^ Previous works have demonstrated that UACR lowering mediates up to 87% of the finerenone treatment effect on kidney outcomes in T2D.^20^^,^^22^ Beyond its effects on albuminuria, finerenone, similar to RAS inhibitors and SGLT2 inhibitors, may induce an acute, reversible reduction in eGFR after treatment initiation. This initial eGFR “dip” is thought to reflect favorable hemodynamic changes that reduce intraglomerular pressure and has been associated with sustained preservation of kidney function and improved long-term kidney outcomes in patients with T2D and CKD.^23^^,^^24^

However, the clinical significance and safety implications of the acute eGFR response to finerenone in people with T1D remains uncertain. Therefore, the aim of this FINE-ONE prespecified analysis was to describe acute eGFR changes in response to finerenone, assess whether the different types of acute eGFR change varied according to characteristics at baseline, and investigate the impact of these eGFR changes on safety parameters.

## Methods

### Study Design and Patients

FINE-ONE was a randomized, prospective, double-blind, multicenter, phase 3 study involving people with T1D and CKD (NCT05901831). The design and baseline characteristics have been published previously.^12^^,^^20^ The primary objective of FINE-ONE was to demonstrate whether finerenone was superior to placebo in reducing UACR over 6 months when given in addition to standard-of-care. The trial was conducted in compliance with the principles of the Declaration of Helsinki and in accordance with Good Clinical Practice guidelines. All study participants provided written informed consent.

Eligible individuals were ≥ 18 years of age at screening, with a diagnosis of T1D. Participants were on a stable dose of angiotensin-converting enzyme inhibitor or angiotensin receptor blocker for at least 4 weeks before screening. Other inclusion criteria included a glycated hemoglobin (HbA_1c_) of < 10.0%, an eGFR of ≥ 25 to < 90 ml/min per 1.73 m^2^, and a UACR of ≥ 200 to < 5000 mg/g. Full eligibility criteria have been described previously.^12^

In total, 242 participants were randomized in a 1:1 ratio to finerenone or matching placebo. The starting dose was 10 mg or 20 mg once daily depending on the baseline eGFR, with 10 mg once daily for an eGFR of ≥ 25 to < 60 ml/min per 1.73 m^2^ and the higher (target) dose of 20 mg once daily for an eGFR of ≥ 60 ml/min per 1.73 m^2^. After 30 days, study medication could be increased to the target dose of 20 mg if the serum potassium concentration was ≤ 4.8 mmol/l and eGFR decrease was < 30% of the previously measured eGFR value. Scheduled study visits during the planned treatment period occurred at randomization and months 1, 3, and 6. The follow-up visit was scheduled 30 days after the last dose of study intervention. Demographic characteristics and medical history were recorded at screening. Blood and urine samples were collected at study visits to assess UACR, eGFR, and potassium, with UACR assessed at baseline, month 3, month 6, and follow-up.

### Outcomes

The primary outcome of this prespecified analysis was the acute change in eGFR from baseline to month 1 (visit 3), also termed “eGFR dip.” The reversibility of the eGFR change was assessed by determining eGFR after treatment discontinuation during the washout phase from month 6 to month 7. The changes in eGFR at both time points were further investigated across prespecified subgroups, including baseline eGFR (< 45 ml/min per 1.73 m^2^, 45 to < 60 ml/min per 1.73 m^2^, and ≥ 60 ml/min per 1.73 m^2^) and UACR levels (< 300, 300–1000, and > 1000 mg/g), baseline use of diuretics (yes/no), and a history of CVD (yes/no).

The safety implications of the acute change in eGFR were examined by determining the number of TEAEs and serious adverse events, stratified by the magnitude of the initial eGFR change and by treatment arm (finerenone vs. placebo). In addition, we explored the association between the acute change in eGFR at month 1 and changes in efficacy and safety biomarkers, including UACR and serum potassium at 1 month and UACR at 3 months because UACR was not measured after 1 month.

### Patient Subgroups

Participants were categorized into the following 3 “eGFR dip” subgroups based on their month 1 eGFR response: a decline of > 10%, a decline between > 0% and ≤ 10%, and no decline or an increase (≤ 0%) in eGFR, consistent with previously established thresholds.^25^ Baseline characteristics are reported for each eGFR change subgroup. In addition, changes in UACR for each eGFR dip subgroup and according to the tertile of eGFR decline were calculated, as reported in previous analyses.^26^^,^^27^

### Statistical Analysis

This analysis was performed on the safety analysis set, which comprised all randomized participants who received at least 1 dose of finerenone or placebo.^21^ Baseline demographic and clinical characteristics were reported, with continuous variables reported as means (SD) or medians (range) and categorical variables as frequencies and percentages. The change in eGFR from baseline to month 1 (visit 3) was analyzed using a mixed model for repeated measures with the following factors: treatment group, visit, treatment-by-visit interaction, baseline value as a covariate, and baseline value-by-visit interaction. The change in eGFR from month 6 to follow-up was analyzed using analysis of covariance, adjusted for treatment and the eGFR value at month 6. Interaction terms were assessed to determine whether the effect of treatment varied across baseline subgroups of interest. Adverse events were reported by the aforementioned eGFR dip groups. Adverse events that started or worsened after the first dose of study intervention and up to 3 days after any temporary or permanent interruption of study intervention were considered as TEAEs. Logistic regression, using both univariable and multivariable stepwise backward selection (based on the Akaike information criterion), was used to identify baseline factors associated with an acute eGFR dip > 10%. To evaluate the relationship between the acute eGFR dip and early changes in UACR and serum potassium, we reported changes in these biomarkers by predefined eGFR dip groups as means with 95% CIs for linear parameters and as geometric mean percentage changes with 95% CIs for log-transformed UACR values. All analyses were conducted using R software (version 4.5.1; R Foundation for Statistical Computing, Vienna, Austria).

## Results

### Study Population Characteristics

In the FINE-ONE trial, 242 adults with T1D and CKD were randomly assigned to receive finerenone or matching placebo.^21^ Of these, 241 participants (119 receiving finerenone and 122 receiving placebo) received at least 1 dose and were included in the safety analysis set and, therefore, in this eGFR dip analysis. Baseline characteristics of FINE-ONE trial participants according to eGFR dip subgroups at month 1 are shown in Table 1. Information on eGFR change at month 1 was available for 234 participants. The distribution of eGFR change at month 1 by treatment arm is shown in Figure 1. The majority of participants experienced no change or an increase in eGFR (finerenone, 51; placebo, 51), followed by > 0%–≤ 10% eGFR dip (finerenone, 32; placebo, 43) and > 10% eGFR dip (finerenone, 33; placebo, 24). The baseline mean eGFR was highest in participants with a > 10% eGFR dip (64.6 ml/min per 1.73 m^2^), followed by those with a > 0%–≤ 10% eGFR dip (61.1 ml/min per 1.73 m^2^) and those with no change or an increase in eGFR (55.3 ml/min per 1.73 m^2^). Participants with >10% eGFR decline also had the highest baseline UACR and body mass index (BMI; calculated as weight in kg divided by height in m^2^). Baseline blood pressure, serum potassium, and HbA_1c_ were similar across groups. Diuretic use was most prevalent in the eGFR increase cohort at 39%, followed by 35% and 32% use in the > 0%–≤ 10% and > 10% eGFR dip cohorts, respectively.

**Table 1:** Baseline characteristics by acute eGFR change from baseline at month 1 in eGFR categories (safety analysis set)

Characteristic	Acute eGFR change subgroup		
	eGFR increase^a^ (*n* = 102)	> 0%–≤ 10% eGFR reduction (*n* = 75)	> 10% eGFR reduction (*n* = 57)
Treatment group, *n* (%)			
Finerenone	51 (50.0)	32 (42.7)	33 (57.9)
Placebo	51 (50.0)	43 (57.3)	24 (42.1)
Age, yrs, mean (SD)	50.6 (14.1)	52.2 (13.9)	51.2 (12.5)
Male, *n* (%)	70 (68.6)	50 (66.7)	36 (63.2)
Race and ethnicity, *n* (%)			
White	66 (64.7)	59 (78.7)	43 (75.4)
Asian	25 (24.5)	12 (16.0)	11 (19.3)
Black	8 (7.8)	4 (5.3)	2 (3.5)
Other	3 (2.9)	0	1 (1.8)
Systolic blood pressure, mm Hg, mean (SD)	134.2 (17.4)	136.0 (16.1)	135.9 (16.6)
Diastolic blood pressure, mm Hg, mean (SD)	78.0 (10.3)	76.8 (11.6)	78.7 (9.5)
Duration of diabetes, yr, mean (SD)	30.3 (13.6)	32.9 (14.2)	32.6 (14.6)
Age at diabetes onset, yr, mean (SD)	20.8 (14.2)	19.9 (12.8)	19.1 (13.9)
HbA_1c_, %, mean (SD)	7.6 (1.0)^b^	7.6 (1.2)	7.7 (1.0)
Serum potassium, mmol/l, mean (SD)	4.6 (0.5)	4.6 (0.4)	4.5 (0.4)
eGFR, ml/min per 1.73 m^2^, mean (SD)	55.3 (18.2)	61.1 (20.2)	64.6 (18.2)
UACR, mg/g, median (min–max)	563.6 (92.8–4845.2)	518.9 (76.9–3406.9)	591.4 (130.8–3100.2)
Weight, kg (SD)	78.4 (18.8)^c^	79.6 (20.3)^c^	86.5 (23.5)
BMI^d^, kg/m^2^, mean (SD)	26.5 (5.3)^c^	27.2 (5.5)^c^	29.7 (7.2)
Medical history findings of interest, *n* (%)			
Cardiovascular disease	26 (25.5)	20 (26.7)	11 (19.3)
Hypertension	85 (83.3)	63 (84.0)	51 (89.5)
Heart failure	3 (2.9)	1 (1.3)	2 (3.5)
Baseline medications, *n* (%)			
ACEi	42 (41.2)	37 (49.3)	27 (47.4)
ARB	59 (57.8)	37 (49.3)	30 (52.6)
Diuretics	40 (39.2)	26 (34.7)	18 (31.6)

ACEi, angiotensin-converting enzyme inhibitor; ARB, angiotensin receptor blocker; BMI, body mass index; eGFR, estimated glomerular filtration rate; HbA_1c_, glycated hemoglobin; SD, standard deviation; UACR, urine albumin-to-creatinine ratio; yr, year(s).

Subgroup and treatment group populations include only participants with available eGFR data at month 1 (visit 3). The total number of participants was 234.

a Category includes participants with no change in eGFR.

b Two missing values.

c One missing value.

d Calculated as weight in kg divided by height in m^2^.

**Figure 1:**
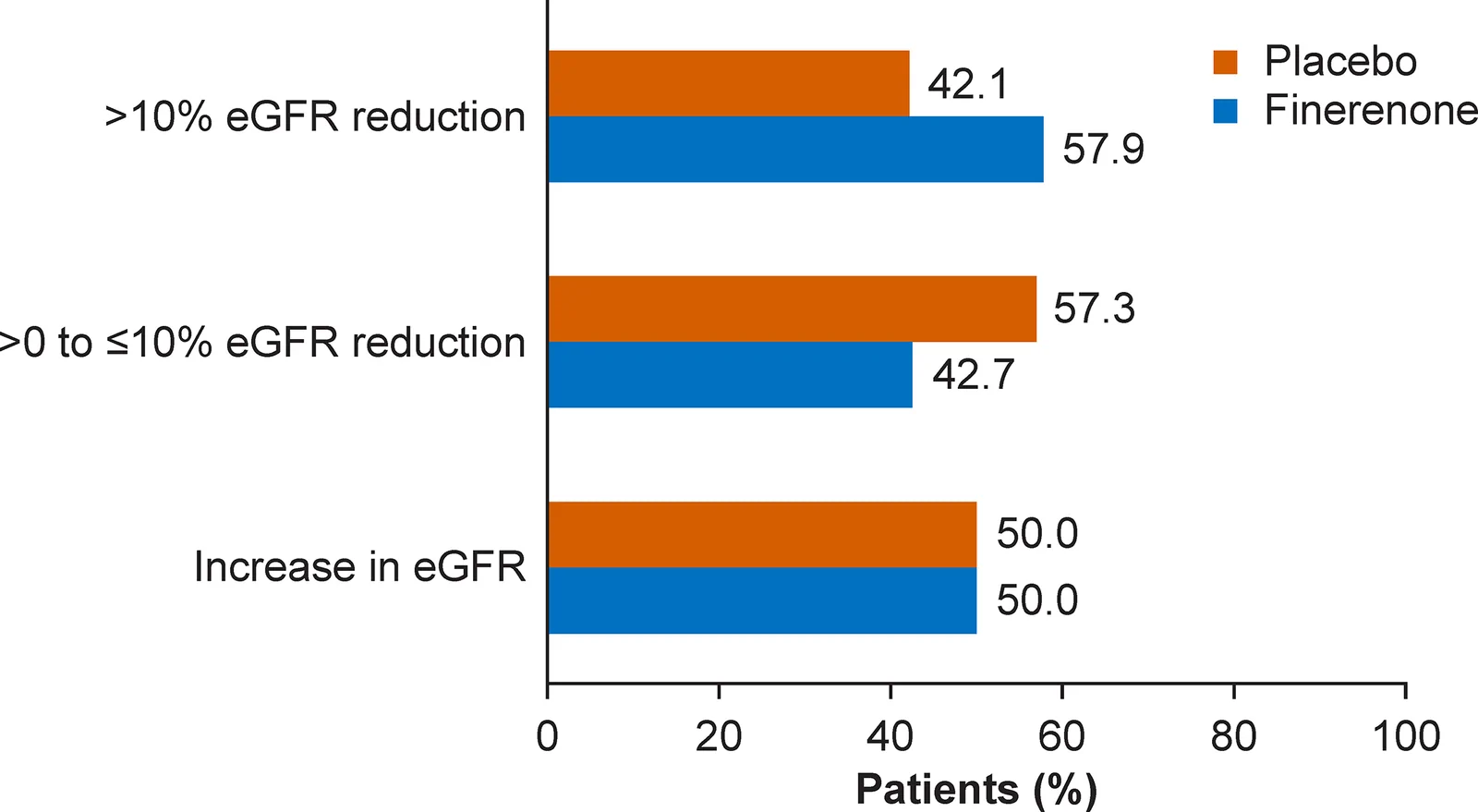
Distribution of eGFR change at month 1 by treatment arm. The increase in eGFR category includes participants with no change in eGFR. Subgroup and treatment group populations include only participants with available eGFR data at month 1 (visit 3). The total number of participants was 234. eGFR, estimated glomerular filtration rate.

### Acute Changes in eGFR and Reversibility in eGFR After Treatment Discontinuation

In the overall population, the acute change in eGFR from baseline to 1 month postrandomization was greater with finerenone (−2.78 ml/min per 1.73 m^2^ [95% CI, −4.15 to −1.40]) versus placebo (−0.78 ml/min per 1.73 m^2^ [95% CI, −2.22 to 0.66]), with a treatment difference of –1.99 ml/min per 1.73 m^2^ (95% CI, –3.97 to –0.01 [*P* = 0.049]). The acute eGFR dip with finerenone compared with placebo was generally consistent according to baseline eGFR (< 45, 45 to < 60, and ≥ 60 ml/min per 1.73 m^2^) and UACR (< 300, 300–1000, and > 1000 mg/g), as well as baseline history of CVD and use of a diuretic (Figure 2). When participants were grouped according to acute eGFR change at month 1, the mean change was 3.57 ml/min per 1.73 m^2^ (95% CI, 2.58–4.56) and 6.12 ml/min per 1.73 m^2^ (95% CI, 4.53–7.71) in the no decline or an increase group, –3.56 ml/min per 1.73 m^2^ (95% CI, –4.28 to –2.83) and –3.33 ml/min per 1.73 m^2^ (95% CI, –3.91 to –2.74) in the > 0%–≤ 10% decline group and –11.85 ml/min per 1.73 m^2^ (95% CI, –14.46 to –9.24) and –10.87 ml/min per 1.73 m^2^ (95% CI, –13.21 to –8.53) in the > 10% decline group for finerenone versus placebo, respectively.

**Figure 2:**
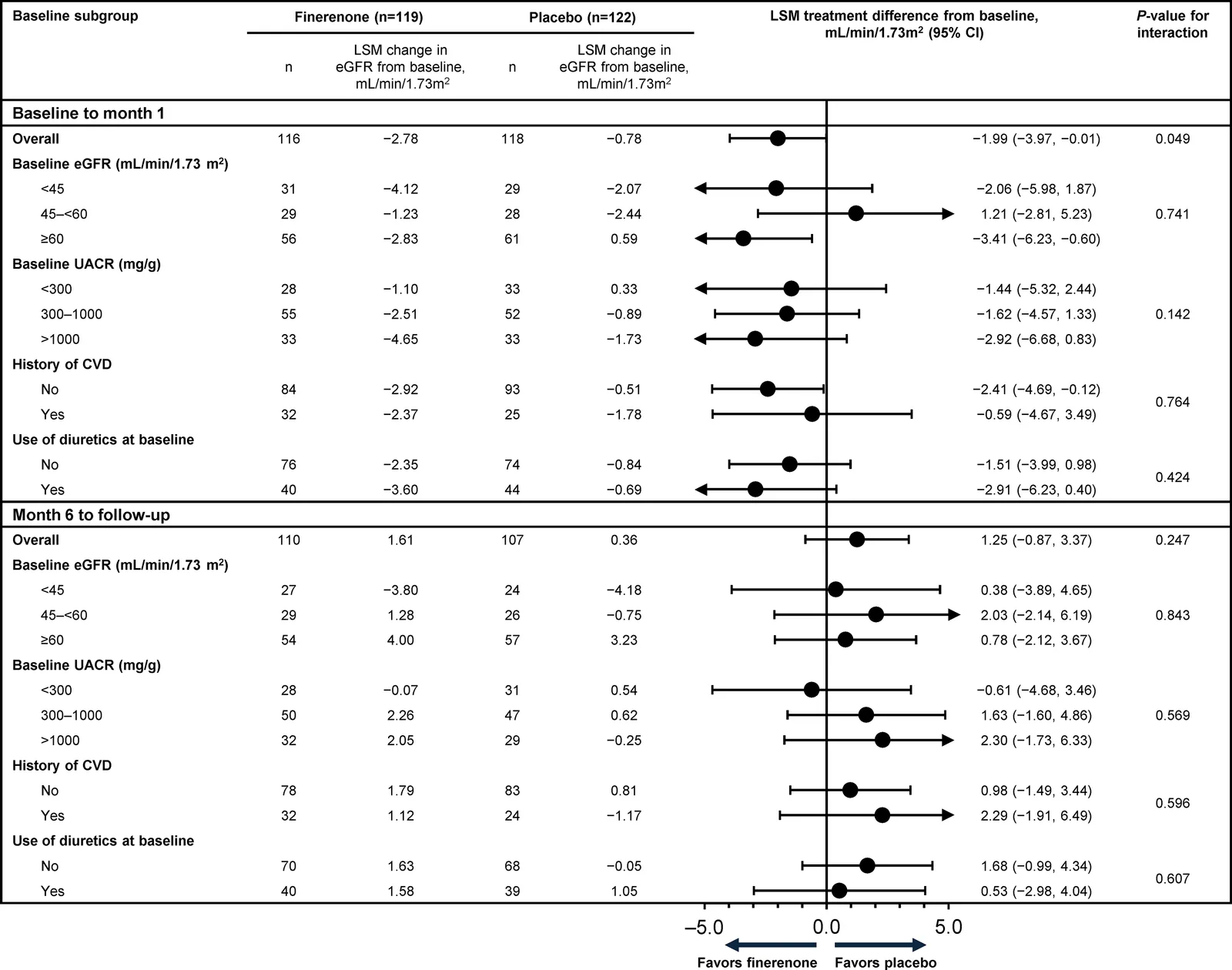
Finerenone versus placebo: mean change in eGFR according to baseline eGFR and UACR subgroups and history of CVD and diuretic use. Subgroup and treatment group populations include only participants with available eGFR data at month 1 (visit 3). The total number of participants was 234 (*n* = 116 for finerenone and *n* = 118 for placebo). CI, confidence interval; CVD, cardiovascular disease; eGFR, estimated glomerular filtration rate; LSM, least squares mean; UACR, urine albumin-to-creatinine ratio.

From end of treatment to follow-up, the increase in eGFR was greater with finerenone (1.61 ml/min per 1.73 m^2^ [95% CI, 0.12 to 3.09]) compared with placebo (0.36 ml/min per 1.73 m^2^ [95% CI, −1.15 to 1.86]), although this did not reach statistical significance (treatment difference: 1.25 ml/min per 1.73 m^2^; 95% CI, –0.87 to 3.37 [*P* = 0.247]). The eGFR dip was reversible after treatment discontinuation overall and across eGFR, UACR, history of CVD, and diuretic use subgroups (Figure 2).

### Baseline Factors Associated With eGFR Dip > 10%

In univariable analyses for all treatment arms, baseline BMI, baseline body weight, and baseline eGFR were associated with higher odds of experiencing a > 10% eGFR dip from baseline to month 1 (Table 2). In the multivariable backward selection model, treatment arm, baseline BMI, baseline eGFR, and baseline sodium were retained. BMI (odds ratio [OR], 1.09 [95% CI, 1.04–1.15] [*P* = 0.001]) and baseline eGFR (OR, 1.02 [95% CI, 1.00–1.04] [*P* = 0.02]) were significantly associated with higher odds of a > 10% eGFR dip at month 1. The odds ratio associated with finerenone treatment was 1.44 (95% CI, 0.76–2.74) and each mmol/l increment in baseline sodium was associated with higher odds of experiencing a > 10% eGFR dip (OR, 1.09 [95% CI, 0.97–1.22]).

**Table 2:** Univariable and multivariable regression analyses for a > 10% acute eGFR reduction at month 1 in all treatment arms (*n* = 225)

Baseline characteristic	Univariable analysis			Multivariable analysis		
	OR	95% CI	*P-*value	OR	95% CI	*P*-value
Age, yr	1.00	0.98–1.02	0.95	–	–	–
Male	0.89	0.48–1.70	0.72	–	–	–
Treatment (finerenone vs. placebo)	1.52	0.83–2.82	0.18	1.44	0.76–2.74	0.26
Pooled race^a^						
Asian	–	–	–	–	–	–
Black	0.61	0.09–2.75	0.56	–	–	–
White	1.20	0.57–2.65	0.64	–	–	–
Other	1.12	0.05–9.81	0.92	–	–	–
BMI^b^, kg/m^2^	1.08	1.03–1.14	0.002	1.09	1.04–1.15	0.001
Baseline body weight, kg	1.02	1.01–1.03	0.008	–	–	–
SBP, mm Hg	1.00	0.99–1.02	0.63	–	–	–
DBP, mm Hg	1.01	0.98–1.04	0.38	–	–	–
eGFR, ml/min per 1.73 m^2^	1.02	1.00–1.04	0.02	1.02	1.00–1.04	0.02
UACR^c^	1.24	0.87–1.77	0.23	–	–	–
Sodium, mmol/l	1.06	0.96–1.18	0.25	1.09	0.97–1.22	0.15
Serum potassium, mmol/l	0.63	0.30–1.31	0.22	–	–	–
HbA_1c_, %	1.08	0.81–1.45	0.59	–	–	–
Uric acid, μmol	0.85	0.68–1.05	0.15	–	–	–
Diuretic medication	0.78	0.40–1.46	0.44	–	–	–
Duration of T1D, yr	1.00	0.98–1.03	0.68	–	–	–
History of CVD (medical history)	1.62	0.78–3.64	0.22	–	–	–
History of hypertension	0.60	0.22–1.46	0.29	–	–	–
History of heart failure	0.65	0.12–4.82	0.63	–	–	–

BMI, body mass index; CI, confidence interval; CVD, cardiovascular disease; DBP, diastolic blood pressure; HbA_1c_, glycated hemoglobin; OR, odds ratio; SBP, systolic blood pressure; T1D, type 1 diabetes; UACR, urine albumin-to-creatinine ratio; yr, year(s).

Univariable logistic regression analyses include only participants with complete (nonmissing) data for all predictors considered.

a The reference population was Asian.

b Calculated as weight in kg divided by height in m^2^.

c UACR (mg/g) was log-transformed before entry into the regression models. The odds ratio corresponds to a one-unit increase on the log scale.

In a univariable analysis with the finerenone treatment arm only, none of the clinical parameters were associated with > 10% in eGFR at month 1 (Supplementary Table S1). In the multivariable backward selection model, there were no associations between clinical parameters and > 10% dip in the eGFR.

### Safety

TEAEs are shown in Table 3. Overall, the proportions of TEAEs were similar between the finerenone and placebo groups, although hyperkalemia was more common with finerenone (9.1%–12.5%) than with placebo (0%–5.9%) across the eGFR change subgroups. The incidence of treatment-emergent serious adverse events was similar between treatment arms within each eGFR category, and no deaths were reported.

**Table 3:** Safety by acute eGFR change category and treatment arm (safety analysis set)

*n* (%)	Acute eGFR change subgroup					
	eGFR increase^a^		> 0%–≤ 10% eGFR reduction		> 10% eGFR reduction	
	Finerenone (*n* = 51)	Placebo (*n* = 51)	Finerenone (*n* = 32)	Placebo (*n* = 43)	Finerenone (*n* = 33)	Placebo (*n* = 24)
Any treatment-emergent AE	21 (41.2)	22 (43.1)	18 (56.2)	23 (53.5)	16 (48.5)	12 (50.0)
Intervention related	7 (13.7)	8 (15.7)	5 (15.6)	1 (2.3)	6 (18.2)	3 (12.5)
Leading to permanent discontinuation	1 (2.0)	0	1 (3.1)	1 (2.3)	0	0
Any treatment-emergent SAE	6 (11.8)	6 (11.8)	5 (15.6)	3 (7.0)	2 (6.1)	4 (16.7)
Intervention related	1 (2.0)	0	1 (3.1)	0	0	0
Leading to permanent discontinuation	1 (2.0)	0	1 (3.1)	0	0	0
Requiring hospitalization	6 (11.8)	4 (7.8)	4 (12.5)	3 (7.0)	1 (3.0)	3 (12.5)
Life-threatening^b^	2 (3.9)	0	1 (3.1)	1 (2.3)	0	0
Death	0	0	0	0	0	0
Any hyperkalemia (treatment-emergent AESI)^c^	5 (9.8)	3 (5.9)	4 (12.5)	0	3 (9.1)	1 (4.2)
Any serious hyperkalemia^c^^,^^d^	1 (2.0)	0	1 (3.1)	0	0	0

AE, adverse event; AESI, adverse event of special interest; eGFR, estimated glomerular filtration rate; SAE, serious adverse event.

Subgroup and treatment group populations include only participants with available eGFR data at month 1 (visit 3); the total number of participants was 234.

a Category includes participants with no change in eGFR.

b None of the life-threatening treatment-emergent SAEs were related to study drug according to the investigators.

c Hyperkalemia includes investigator-reported AEs with the Medical Dictionary for Regulatory Activities terms hyperkalemia and blood potassium increased.

d All cases of serious hyperkalemia were in the finerenone group.

### Association Between eGFR Dip and Specified Outcomes

The acute changes in eGFR (eGFR increase, > 0–≤ 10% and > 10% dip) at month 1 postrandomization in response to finerenone and their association with UACR are shown in Table 4 and Figure 3. Greater acute eGFR decline with finerenone was associated with progressively larger reductions in UACR (measured at month 3), with mean percentage changes increasing from −23.6% to −36.7% (Table 4 and Figure 3a). These changes in UACR were similar when acute changes in eGFR were categorized by tertiles (Figure 3b). In the placebo group, UACR change ranged from 1.5% to −32.2% as the magnitude of eGFR dip became greater (Table 4 and Figure 3a). Serum potassium levels increased with larger eGFR dips in the finerenone and placebo groups (Table 4).

**Table 4:** Changes in UACR and serum potassium from baseline by acute eGFR change subgroups

Measure	Acute eGFR change from baseline at month 1					
	Finerenone			Placebo		
	eGFR increase^a^ (*n* = 51)	0%–≤ 10% eGFR reduction (*n* = 32)	> 10% eGFR reduction (*n* = 33)	eGFR increase^a^ (*n* = 51)	0%–≤ 10% eGFR reduction (*n* = 43)	> 10% eGFR reduction (*n* = 24)
UACR^b^	−23.6 (–33.8, –11.9)	−34.0 (−45.3, −20.4)	−36.7 (−48.8, −21.7)	1.5 (−13.7, 19.4)	−11.3 (−25.0, 4.9)	−32.2 (−43.9, −18.1)
Serum potassium	0.03 (−0.07, 0.12)	0.16 (0.01, 0.32)	0.30 (0.13, 0.47)	0.05 (−0.08, 0.17)	0.03 (−0.10, 0.16)	0.23 (0.10, 0.36)

CI, confidence interval; eGFR, estimated glomerular filtration rate; UACR, urine albumin-to-creatinine ratio.

Changes in UACR from baseline to Month 3 are expressed as mean percentage change (95% CI). Changes in serum potassium are expressed as mean (95% CI).

a Category includes participants with no change in eGFR.

b Data were not available for 5 participants in the finerenone group and 5 participants in the placebo group.

**Figure 3:**
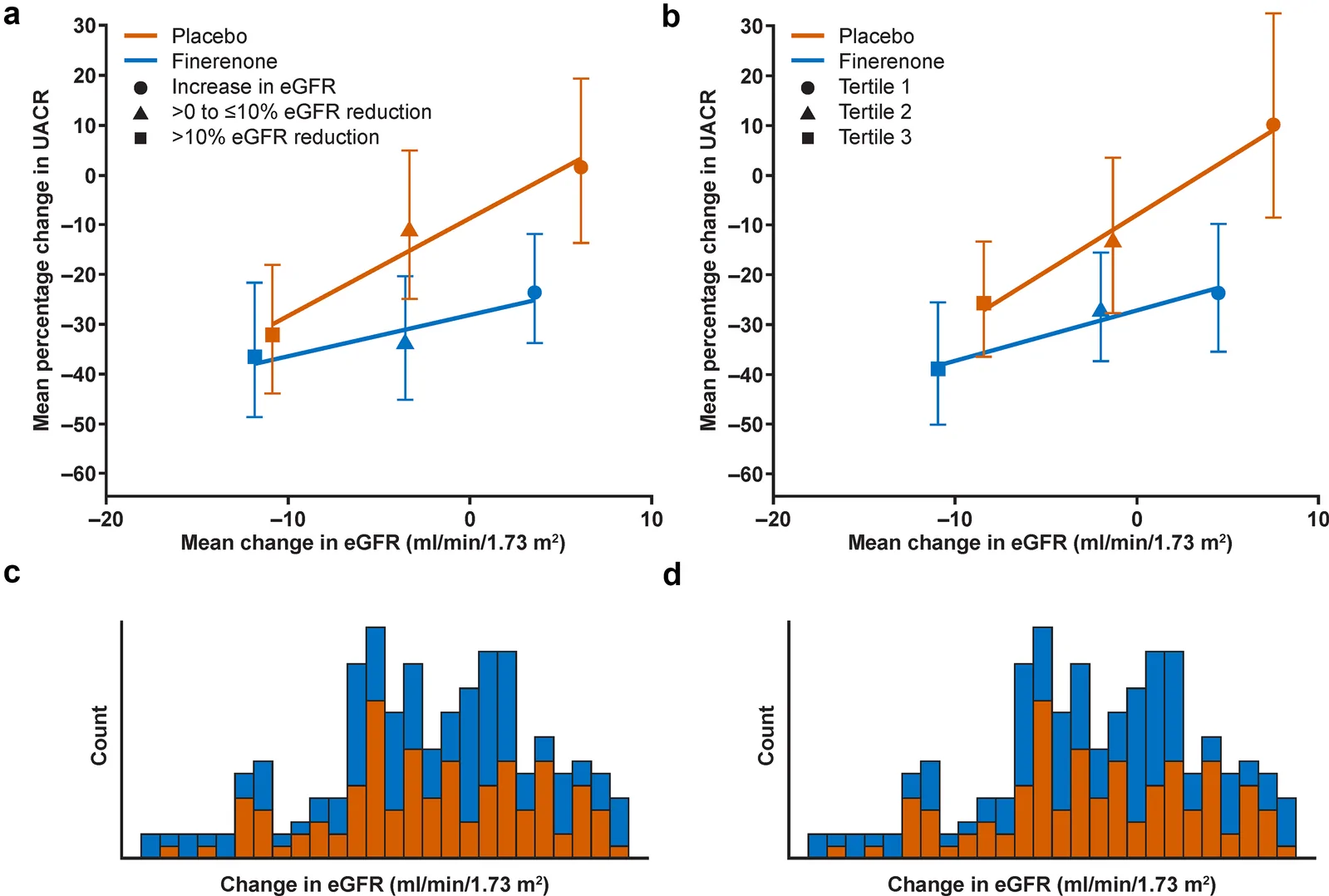
Relationship between mean change in eGFR at month 1 and mean change in UACR at month 3 for each (a) eGFR dip subgroup (eGFR increase,^a^ > 0% to ≤ 10% reduction, > 10% reduction) and (b) eGFR tertile; (c) and (d) show respective frequency histograms for changes in eGFR. Lines in the upper panels represent least-squares linear fits through the subgroup-level means (mean eGFR vs. geometric mean UACR) within each treatment arm and should not be interpreted as an individual-level relationship between acute eGFR change and change in UACR. ^a^Category includes participants with no change in eGFR. eGFR, estimated glomerular filtration rate; UACR, urine albumin-to-creatinine ratio.

## Discussion

In this prespecified analysis of the FINE-ONE trial, which assessed UACR reduction in participants with T1D and CKD, finerenone was associated with an initial decline in eGFR from baseline to month 1. The acute eGFR decline was expected because it was previously described in people with T2D and CKD treated with finerenone.^23^ It was partially reversible after treatment discontinuation at follow-up. The acute changes in eGFR were consistent across prespecified subgroups, including baseline eGFR, UACR, history of CVD, and concomitant diuretic use. Finerenone was well tolerated, with a generally consistent safety profile across eGFR dip categories.

In diabetic kidney disease, maladaptive glomerular hyperfiltration and elevated intraglomerular pressure contribute to progressive kidney injury and albuminuria.^11^^,^^28^ The role of physiological factors as key contributors to disease progression is supported by the efficacy of therapies that attenuate intraglomerular pressure, such as finerenone,^29^, ^30^, ^31^ SGLT2 inhibitors,^6^, ^7^, ^8^ and RAS inhibitors,^32^, ^33^, ^34^ which are associated with long-term preservation of eGFR. Clinically, the reduction in intraglomerular pressure is reflected by an acute and reversible decline in eGFR shortly after initiation of 1 of these agents. The mechanisms responsible for the reduction of glomerular hypertension are likely via effects on the glomerular microvasculature and tubuloglomerular feedback. nsMRAs and RAS blockers induce pre- and postglomerular arteriolar effects,^35^^,^^36^ whereas SGLT2 inhibitors promote proximal tubular natriuresis, restoring tubuloglomerular feedback and resulting in predominant afferent arteriolar vasoconstriction and reduced glomerular hypertension in experimental models.^11^ The acute reduction in eGFR with finerenone in this study was partially reversible upon treatment discontinuation, as evidenced by the subsequent increase in eGFR at follow-up. The early eGFR decline in response to finerenone may serve as a surrogate marker of intraglomerular pressure reduction in people with T1D and CKD, reflecting hemodynamic pathways associated with kidney protection similar to those in T2D. In the present analysis, greater acute decline in eGFR was associated with progressively larger reductions in UACR. This association may in part reflect the shared hemodynamic effects that reduce intraglomerular pressure, resulting in both an initial decline in eGFR and reduced albuminuria.^35^, ^36^, ^37^ However, available evidence suggests that the relationship is unlikely to be explained entirely by hemodynamic mechanisms. In the CONFIDENCE trial, mediation analyses demonstrated that acute eGFR changes accounted for only 5.2% of the additional UACR reduction observed with the addition of finerenone to empagliflozin,^38^ suggesting that nonhemodynamic mechanisms, including antiinflammatory and antifibrotic effects related to mineralocorticoid receptor antagonism, may contribute substantially to lowering of albuminuria.^37^ Notably, participants in FINE-ONE trial did not receive background SGLT2 inhibitors like those in CONFIDENCE trial, and further studies are needed to better elucidate the relationship between acute eGFR changes and UACR reduction with finerenone.

Consistent with the mechanism of reducing glomerular hypertension, acute eGFR reduction after treatment initiation was not associated with adverse outcomes in secondary analyses of seminal SGLT2 inhibitor trials.^39^ Moreover, participants experiencing an acute eGFR decline > 10% exhibited slower subsequent annual eGFR decline compared with those who had a smaller (> 0%–≤ 10%) or no initial decline.^39^ The preservation of treatment effects stratified by acute eGFR decline with finerenone has also been demonstrated in prespecified analyses of the FIDELITY^23^ and FINEARTS-HF^40^ trials. In the FIDELITY analysis, the benefits of finerenone on the composite kidney (kidney failure, a sustained decrease of eGFR ≥ 57% from baseline, or kidney disease death) and cardiovascular outcomes (cardiovascular death, nonfatal myocardial infarction, nonfatal stroke, or hospitalization for heart failure) were maintained across groups defined by acute eGFR decline (> 10% vs. > 0%–≤ 10%).^23^ In FINEARTS-HF, the benefits of finerenone on total heart failure events and cardiovascular death were consistent across the range of change in eGFR stratified by those with ≥ 15% versus no decline in eGFR.^40^ Across both analyses, finerenone showed efficacy and comparable adverse event rates irrespective of the magnitude of acute eGFR dip. Although FINE-ONE trial was not powered to assess cardiovascular and kidney event outcomes, like FIDELITY and FINEARTS-HF trials, the effects of finerenone on lowering of UACR as an efficacy outcome were preserved in the > 10% dip group, and safety was similar across eGFR change subgroups.

Finerenone and RAS inhibitors are guideline-directed therapies for the management of CKD in T2D.^41^ Due to the mechanisms of action of and RAS inhibitors, people with CKD treated with these agents have potential to develop hyperkalemia adverse events. In the present analysis, serum potassium increased concordantly with the magnitude of the acute eGFR dip. These findings are in accordance with a prespecified analysis of the FIDELITY study, in which larger eGFR declines were associated with higher potassium concentrations. Among individuals with an eGFR decline > 10% in FIDELITY, treatment discontinuation due to hyperkalemia occurred in 1.7% of the finerenone group versus 0.7% in the placebo group.^23^ These results indicate that although serum potassium rises with greater acute eGFR dip, clinically significant hyperkalemia and treatment discontinuation remain infrequent. Accordingly, closer monitoring of serum potassium may be warranted in patients with larger eGFR dip. To better understand factors associated with acute eGFR decline, a multivariable-adjusted analysis was conducted. In the overall trial cohort, baseline BMI and eGFR emerged as independent predictors, whereas treatment assignment and baseline serum sodium did not. The prespecified FIDELITY analysis in T2D identified age, higher eGFR, UACR, systolic blood pressure at baseline, diuretic or β-blocker use, and finerenone use as predictors of an acute eGFR dip at month 1.^23^ The more limited set of predictors identified in the present study may reflect the smaller sample size in the FINE-ONE trial, as well as differences in baseline comorbidities and concomitant medication use.

The strength of this analysis is that it is the first study to elucidate acute eGFR changes in response to finerenone in participants with T1D and CKD. Nevertheless, these findings should be interpreted in the context of some limitations. First, the FINE-ONE trial was not powered to detect differences in the effect stratified by eGFR dip, a limitation compounded by missing data for the acute eGFR change at 30 days postrandomization. As eGFR dip status was defined using eGFR response at month 1, comparisons between dip categories are no longer protected by randomization and may be subject to confounding. Second, as the primary outcome is a surrogate assessed over a short follow-up, the study cannot draw conclusions regarding long-term clinical outcomes for CKD with finerenone in this population with T1D. Finally, the predominantly White and male composition of the trial cohort may limit the generalizability of these findings to more diverse populations.

In conclusion, the magnitude of acute eGFR decline after initiation of finerenone in people with T1D and CKD aligned with the expected hemodynamic effects, which can be interpreted as a reflection of reduced glomerular hypertension. The eGFR dip appeared well tolerated without unexpected safety signals.

## Appendix

### List of FINE-ONE Investigators

**Canada:** David Cherney, Toronto General Hospital - University Health Network, Renal Physiology Laboratory, Toronto; Richard Dumas, Centre de Recherche Clinique de Laval, Laval; Peter Senior, Alberta Diabetes Institute - Clinical Research Unit (CRU), Edmonton; Ronald Sigal, Richmond Road Diagnostic and Treatment Centre - Clinical Trials Unit (RRDTC), Calgary; Jean-Francois Yale, McGill University Health Centre (MUHC) - Research Institute (RI) - McConnell Centre for Innovative Medicine (CIM), Montreal. **China:** Xinhuan An, Zhongshan People's Hospital, Zhongshan; Xiaoling Cai, Peking University People's Hospital, Beijing; Shiwei Liu, Shanxi Bethune Hospital, Taiyuan; Yibing Lu, Nanjing Medical University (NMU) - The Second Affiliated Hospital, Nanjing; Li Mao, Huai’an First People's Hospital, Nanjing Medical University, Huai’an; Yufan Wang, Shanghai Jiao Tong University (SJTU) - Shanghai General Hospital (Shanghai First People's Hospital) - Northern Location, Shanghai; Zhongjing Wang, The Central Hospital of Wuhan, Tongji Medical College, Huazhong University of Science and Technology, Wuhan; Yao Ming Xue, Southern Medical University - Nanfang Hospital (Southern Hospital), Guangzhou; Jialin Yang, Central Hospital of Minhang District, Shanghai; Guoyue Yuan, Affiliated Hospital of Jiangsu University, Zhenjiang; Min Zhang, Zhongshan Hospital Fudan University, Qingpu Branch, Shanghai; Cheng Zhifeng, The 4th Affiliated Hospital of Harbin Medical University, Harbin. **Denmark:** Jesper Bech, Region Midtjylland, Regionshospitalet Godstrup - Nephrology Department, Herning; Claus Juhl, Hospital of South West Jutland, Department of Endocrinology Research, Esbjerg; Ulrik Pedersen-Bjergaard, Capital Region, Nordsjaellands Hospital - Hillerod - Endocrinology Ambulatory Research Unit, Hillerod; Peter Rossing, Steno Diabetes Center Copenhagen, Herlev - Clinical and Translational Research Department, Herlev; Karoline Schousboe, Odense University Hospital, Odense - Endocrinology Department, Odense; Peter Vestergaard, Region Nordjylland, Aalborg University Hospital - Endocrinology Department, Aalborg. **Germany:** Spasija Parizova, Ruhr-Universitaet Bochum - Herz und Diabeteszentrum NRW (Heart and Diabetes Center), Bad Oeynhausen; Thomas Schurholz, InnoDiab Forschung GmbH, Essen. **Italy:** Agostino Consoli, Università degli Studi "G. D'Annunzio" di Chieti – Endocrinologia, Chieti; Paolo Fiorina, ASST Fatebenefratelli Sacco Ospedale Sacco - Malattie Endocrine e Diabetologia, Milan; Edoardo Mannucci, Careggi University Hospital - Diabetologia e Malattie del Metabolismo, Firenze; Uberto Pagotto, Azienda Ospedaliero-Universitaria Di Bologna IRCCS Policlinico Sant'Orsola - Endocrinologia e prevenzione e cura del diabete, Bologna; Dario Pitocco, Fondazione Policlinico Universitario Agostino Gemelli IRCCS - UOSD Diabetologia, Rome; Francesco Romeo, Azienda Sanitaria Locale di Chieri, TO5 - SC Diabetologia Territoriale, Chieri; Emanuela Setola, IRCCS Ospedale San Raffaele, Diabetologia Department - Cardio-Metabolic and Clinical Trials Unit, Milan; Roberto Trevisan, ASST Papa Giovanni XXIII, Ospedale di Bergamo - SC Malattie Endocrine-Diabetologia, Bergamo; Anna Maria Veronelli, ASST Santi Paolo e Carlo, San Paolo Hospital - Haemostasis and Thrombosis Department, Milan. **South Korea:** Jae Hyeon Kim, Samsung Medical Center - Oncology Department, Seoul; Sin Gon Kim, Korea University Anam Hospital, Seoul; Soo Heon Kwak, Seoul National University Hospital, Seoul; Byung Wan Lee, Severance Hospital, Yonsei University Health System, Seoul; Woo Je Lee, Asan Medical Center - Oncology Department, Seoul. **Spain:** Antonio Jesus Amor Fernandez, Hospital Clinic de Barcelona, Instituto Clinic de Enfermedades Digestivas y Metabolicas - Endocrinologia y Nutricion, Barcelona; Miguel Angel Brito Sanfiel, Hospital Universitario Puerta De Hierro De Majadahonda – Endocrinologia, Madrid; Olga Gonzalez Albarran, Hospital Gregorio Maranon, Endocrinology Department, Madrid; Cristóbal Morales Portillo, Hospital Nisa Sevilla Aljarafe – Endocrinologia, Seville; Alfonso Soto Gonzalez, Gerencia de Gestion Integrada A Coruna, Department of Endocrinology and Nutrition, A Coruña; Francisco Jose Tinahones Madueno, Hospital Universitario Virgen De La Victoria – Endocrinologia, Malaga. **United Kingdom:** Gemma Currie, NHS Greater Glasgow and Clyde (NHSGGC) - Glasgow Royal Infirmary (GRI), Glasgow; Siân Griffin, Cardiff and Vale University Health Board, University Hospital of Wales - Nephrology and Transplant, Cardiff; Basil Issa, Wythenshawe Hospital, Manchester; Huda Mahmoud, Walsall Healthcare NHS Trust - Manor Hospital, Walsall; Kieran McCafferty, Barts Health NHS Trust - Royal London Hospital - Nephrology, London; Albert Power, North Bristol NHS Trust - Southmead Hospital, Bristol; Nick Selby, University Hospitals of Derby and Burton NHS Foundation Trust - Royal Derby Hospital, Derby. **United States:** Grazia Aleppo, Northwestern University, Feinberg School of Medicine - Division of Endocrinology, Metabolism and Molecular Medicine, Chicago; Radica Alicic, Providence Inland Northwest Health, Spokane; Marina Basina, Stanford Endocrinology Clinic, Stanford; Bruce Bode, Atlanta Diabetes Associates, Atlanta; Maria Caramori, Cleveland Clinic - Main Campus, Cleveland; Michael Dao, National Institute of Clinical Research, Garden Grove; Ayotunde Dokun, Preventive Intervention Center - Endocrinology, Iowa City; Lenita Hanson, Hanson Clinical Research Center, Inc., Port Charlotte; Andrew Henry, Jedidiah Clinical Research, Temple Terrace; William Kaye, Metabolic Research Institute, Inc., West Palm Beach; Klara Klein, University of North Carolina (UNC) - Hospitals Outpatient Center at Eastowne, Chapel Hill; Guido Lastra, University of Missouri Health Care, Columbia; Arvind Madan, Elixia Central Florida, Hollywood; Jany Moussa, Wichita Nephrology Group Pa, Wichita; Jean Park, MedStar Health Research Institute, Washington; John C. Parker, Accellacare, Wilmington; Louis Philipson, UChicago Medicine Kovler Diabetes Center, Chicago; Rodica Pop-Busui, OHSU Physicians Pavilion – Endocrinology, Portland; Minesh Rajpal, Southwest Kidney Institute, PLC, Surprise; Sylvia Rosas, Joslin Diabetes Center, Boston; Maamoun Salam, Washington University School of Medicine in St. Louis, St. Louis; Jay H. Shubrook, Touro University California - Metabolic Research Center, Vallejo; Elias Siraj, EVMS Strelitz Diabetes Center, Norfolk; Carolina Solis-Herrera, UT Health San Antonio - Medical Arts & Research Center – Endocrinology, San Antonio; Tina Thethi, AdventHealth Translational Research Institute, Orlando; Tuan-Huy Tran, Diabetes & Metabolism Associates, Metairie; Guillermo Umpierrez, Grady Memorial Hospital – Endocrinology, Atlanta; Francesco Vendrame, University of Miami Leonard M. Miller School of Medicine (UMMSM) - Peggy and Harold Katz Family Drug Discovery Center, Miami; Mark Warren, Physicians East, P.A. – W. H. Smith – Endocrinology, Greenville; Michelle Welch, Consano Clinical Research, LLC, San Antonio.

## Disclosure

DZIC has received honoraria from Boehringer Ingelheim–Lilly, Merck, AstraZeneca, Sanofi, Mitsubishi-Tanabe, AbbVie, Janssen, Amgen, Bayer, Prometic, Bristol Myers Squibb, Maze, Gilead, CSL Behring, Otsuka, Novartis, Youngene, Lexicon, Inversago, GSK, Biobridge, Vantage, Altimmune, and Novo Nordisk and has received operational funding for clinical trials from Boehringer Ingelheim–Lilly, Merck, Janssen, Sanofi, AstraZeneca, CSL Behring, Lexicon, Novo Nordisk, and Bayer. TWY has received conference and travel support from Lexicon Pharmaceuticals and Boehringer Ingelheim and receives support from the Banting & Best Diabetes Centre Postdoctoral Fellowship. HJLH has received consulting fees from Alexion, Amgen, AstraZeneca, Bayer, Biocity Pharmaceutics, Boehringer Ingelheim, Dimerix, Eli Lilly, Novartis, Novo Nordisk, Roche, and Travere Therapeutics; received research funding from AstraZeneca, Bayer, Boehringer Ingelheim, Janssen, and Novo Nordisk; and received honoraria for lectures from AstraZeneca, Bayer, and Novo Nordisk. He is member of the Kidney Week 2025 Program Committee for the American Society of Nephrology. JBM has received grants from the National Institutes of Health (NIH), Breakthrough T1D, Diamyd, Cour, Viking, Novo Nordisk, and Lilly. She has received royalties from Washington University and consulting fees from Abbott, Bayer, Mannkind, Novo Nordisk, Pfizer, and Vertex. JNB has received consulting fees from AstraZeneca and Bayer. PF reports leadership or fiduciary role in the Italian Society of Diabetes and has received consulting fees from Novo Nordisk, Eli Lilly, Roche, and Amgen. LJ has received consulting fees from Eli Lilly, Novo Nordisk, Merck, Bayer, Sanofi-Aventis, MSD, AstraZeneca, Abbott, Fosun Pharma, Innovent Biologics, Gan & Lee, Sinocare, and Sibionics; received speaker fees from Eli Lilly, Novo Nordisk, Merck, Bayer, Sanofi-Aventis, MSD, AstraZeneca, Abbott, Fosun Pharma, Innovent Biologics, Gan & Lee, Sinocare, and Sibionics; and received support for attending meetings from Novo Nordisk. AP has received honoraria from AstraZeneca, Bayer, and Boehringer Ingelheim and received conference attendance support from AstraZeneca and Bayer. KS has received personal advisory fees from Boehringer Ingelheim, Novo Nordisk, and Sanofi. NMS has received consulting fees from Guard Therapeutics and SERB Pharmaceuticals. ES has received consulting fees from Sanofi, Novo Nordisk, and Eli Lilly. KRT has received grants from the National Institute of Diabetes and Digestive and Kidney Diseases (NIDDK), National Heart, Lung, and Blood Institute, National Center for Advancing Translational Sciences, NIH Director’s Office, Centers for Disease Control and Prevention, Breakthrough T1D, Benaroya Research Institute, Doris Duke Foundation, Travere, Bayer, and Otsuka; received consulting fees from Alnylam, AstraZeneca, Bayer, Boehringer Ingelheim, GSK, Novo Nordisk, Otsuka, ProKidney, and Roche; received speaker fees from AstraZeneca, Bayer, Boehringer Ingelheim, Novo Nordisk, and Travere; Chair of data safety monitoring boards for NIDDK and George Clinical Institute; member of the data safety monitoring board for AstraZeneca; Chair of the Diabetic Kidney Disease Collaborative and Co-chair of the American Society of Nephrology Kidney Week 2025 Program Committee. PR has received grants to Steno Diabetes Center Copenhagen (SDCC) from Astra Zeneca, Bayer, and Novo Nordisk; consulting fees to SDCC from AstraZeneca, Amgen, Bayer, Boehringer Ingelheim, Novartis, Novo Nordisk, and Roche; and study drugs for investigator-initiated studies from Lexicon, Bayer, AstraZeneca, and Novo Nordisk. RL, MB, and PS are employees of Bayer AG, Germany. JR is an employee of Bayer PLC, UK. P-HG has received investigator-initiated research grants from Eli Lilly and Roche; is an advisory board member for AbbVie, Astellas, AstraZeneca, Bayer, Boehringer Ingelheim, Cebix, Eli Lilly, Janssen, Medscape, Merck Sharp & Dohme, Mundipharma, Nestlé, Novartis, Novo Nordisk, and Sanofi; and has received lecture fees from Astellas, AstraZeneca, Bayer, Berlin Chemie, Boehringer Ingelheim, Eli Lilly, Elo Water, Genzyme, Merck Sharp & Dohme, Medscape, Menarini Foundation, Novartis, Novo Nordisk, PeerVoice, Sanofi, and Sciarc. JDS reports no conflict of interest.

## Supplementary Material

Supplementary File (PDF) **Table S1.** Variables associated with > 10% drop in eGFR at month 1: finerenone arm (*N* = 111). CONSORT checklist.

## Footnotes

Supplementary file (PDF)
**Table S1.** Variables associated with > 10% drop in eGFR at month 1: finerenone arm (*N* = 111). CONSORT checklist.
